# Non-destructive, high-content analysis of wheat grain traits using X-ray micro computed tomography

**DOI:** 10.1186/s13007-017-0229-8

**Published:** 2017-11-01

**Authors:** Aoife Hughes, Karen Askew, Callum P. Scotson, Kevin Williams, Colin Sauze, Fiona Corke, John H. Doonan, Candida Nibau

**Affiliations:** 1 The National Plant Phenomics Centre, Institute of Biological, Rural and Environmental Sciences (IBERS), Aberystwyth University, Gogerddan, Aberystwyth, SY23 3EE UK; 2 Present Address: Faculty of Engineering and Environment, University of Southampton, University Road, Southampton, SO17 1BJ UK

**Keywords:** X-ray micro computed tomography, μCT, Image analysis, 3D vision, Grain traits, Wheat, Temperature

## Abstract

**Background:**

Wheat is one of the most widely grown crop in temperate climates for food and animal feed. In order to meet the demands of the predicted population increase in an ever-changing climate, wheat production needs to dramatically increase. Spike and grain traits are critical determinants of final yield and grain uniformity a commercially desired trait, but their analysis is laborious and often requires destructive harvest. One of the current challenges is to develop an accurate, non-destructive method for spike and grain trait analysis capable of handling large populations.

**Results:**

In this study we describe the development of a robust method for the accurate extraction and measurement of spike and grain morphometric parameters from images acquired by X-ray micro-computed tomography (μCT). The image analysis pipeline developed automatically identifies plant material of interest in μCT images, performs image analysis, and extracts morphometric data. As a proof of principle, this integrated methodology was used to analyse the spikes from a population of wheat plants subjected to high temperatures under two different water regimes. Temperature has a negative effect on spike height and grain number with the middle of the spike being the most affected region. The data also confirmed that increased grain volume was correlated with the decrease in grain number under mild stress.

**Conclusions:**

Being able to quickly measure plant phenotypes in a non-destructive manner is crucial to advance our understanding of gene function and the effects of the environment. We report on the development of an image analysis pipeline capable of accurately and reliably extracting spike and grain traits from crops without the loss of positional information. This methodology was applied to the analysis of wheat spikes can be readily applied to other economically important crop species.

**Electronic supplementary material:**

The online version of this article (doi:10.1186/s13007-017-0229-8) contains supplementary material, which is available to authorized users.

## Background

Agriculture is facing major challenges to provide adequate amounts of food in a changing environment. There is a need to produce high-yielding crop varieties under different predicted abiotic stresses. A great deal of progress in generating genomic tools for the major food crops means that the current challenge is to link genetic variation to plant phenotype. Although our ability to analyse phenotype in a comprehensive and automated manner is rapidly developing, we still lack key capacities to analyse phenotypic variation at the population level and thereby dissect the complex genetic and environmental interactions that underpin breeding efforts (reviewed in [1, 2]).

Bread wheat is an important crop in temperate climates, widely used for human consumption and animal feed and a key target in breeding programs. Since domestication some 10,000 years ago in the Fertile Crescent, wheat has become one of the most important food crops worldwide, not only economically but also culturally [3].

Arguably, two of the most important traits during wheat domestication were the increase in grain size and the development of non-shattering seed. Later, during the green revolution, yields were further increased by introducing semi-dwarf alleles with specifically changed plant architecture, including fewer tillers and more compact spikes with more fertile flowers resulting in increased grain number [4]. Despite the observation that variation in grain shape may affect yield and grain quality including milling and nutritional properties [5], the relationship between spike and grain traits has not been systematically studied; one of the main reasons being that the capture of spike-related grain traits has been labour-intensive, involving painstaking documented destruction of the spikes [6, 7].

The drive behind the development of an alternative method was to address one of the main climate-change related challenges in contemporary crop research, namely to understand how yield-related grain properties are affected by increased temperatures and limited water. Transient increases in temperature can have a dramatic effect on yield in wheat, particularly if applied at specific stages during flowering when cells are in the early stages of meiosis and at anthesis [8–11]. Drought also has a significant impact on wheat yield particularly when applied during the reproductive phase [11]. The effect on yield has mainly been attributed to reduced grain number although, for some varieties and at specific growth stages, grain size could increase to compensate for this [9, 10].

Evaluating the effect of stress on grain set and fill traditionally involves threshing the spikes to release the grain, which are then analysed in bulk. The spike is a complex structure in which individual florets are only semi-synchronised [12, 13] therefore threshing may discard developmentally relevant information.

Computer vision techniques using both visible and non-visible part of the light spectrum have been used to help evaluate the effect of biotic and abiotic factors on plant growth and are now starting to be used in physiological breeding programs [1]. These techniques include growth analysis using visible light imaging, infrared thermography, fluorescence analysis, and spectroscopy imaging [1]. Other imaging techniques including magnetic resonance imaging (MRI) and positron emission tomography (PET) have been used to study plant traits but their use is not widespread [14, 15]. This is due to the fact that both MRI and PET scanners tend to only be available in hospitals and medical research centres mainly due to the high cost of this equipment as well as their maintenance requirements. Recently NMR (nuclear magnetic resonance) was used to determine grain weight and composition on a population scale but this was done on loose grain [16]. Despite these advances, techniques to study the effect of stresses on crop yield, and specifically on grain traits in a fast and non-destructive way that retains positional information, are still largely lacking. Combined with controlled environment growth facilities, these imaging capabilities could offer unparalleled precision in dissecting the effect of environment on phenotype.

X-ray micro computed tomography (μCT) is a non-invasive imaging technique based on differential X-ray attenuation by biological material that may offer a cost-effective alternative. The μCT scanner comprises an X-ray source, a sample rotation stage and an X-ray detector. Attenuation of the X-rays as they pass through the sample is correlated with the density and atomic number of the material and is detected by the image detector as a grey value. Rotation of the beam or of the sample allows for these projections to be acquired from different angles that can be reconstructed as an accurate representation or model of the 3D object [17]. Originally developed as a medical diagnostic tool, recent advances in µCT have led to improvements in scan resolution and quality while reducing scanning time, allowing it to be applied to the study of complex plant traits [17]. The capacity to detect and quantify internal structures in a non-invasive and non-destructive way, combined with the ability to automate the process, means that μCT is an attractive approach to study plant traits. High resolution μCT has been successfully used to analyse soil properties, root structure, developing seeds, shoots, developing panicles and leaves [17–25].

However, this approach has not been so widely applied to study general plant traits as might be expected. There are several possible reasons for this. The majority of the μCT hardware and software has been developed and optimised for medical purposes. Most of the available μCT scanners are designed to give high resolution images or to scan large specimens and thus only a few samples can be scanned in a given time. Smaller and bench top scanners are becoming commercially available and overcome some limitations stated above.

At the software level, the available packages have been specifically designed for human biology and material sciences and lack the flexibility necessary to deal with images obtained from different plant organs at different stages of development.

Here, we report on the development of a robust, high-throughput method that allows rapid and accurate feature extraction from μCT images acquired in batches, using a standard benchtop μCT scanner. This method offers many advantages over previously published work [25], and allows not only to quickly and accurately quantify traditional grain traits like number and size, but also determine grain position along the spike which have previously required destructive and time-consuming dissection. To demonstrate the utility of the approach to address typical research questions, spikes resulting from a multiple stress experiment where plants had been grown under different water regimes and exposed to short periods at different temperatures were examined. Whole spike analyses indicated that grain number along the spike and other grain traits, such as volume, are affected by the treatments. This method is provided in an accessible format with usage instructions and sample data. Finally our method can be used to extract similar features from grass inflorescences with very diverse morphologies, demonstrating its flexibility and potential for wider use.

## Methods

### Plant materials

Spring wheat (*Triticum aestivum* cv Paragon) was grown as single plants in compost (3.5L Levington F2) in the greenhouse (day temperature set to 20 °C and night 15 °C, 14 h day length) until the sixth leaf stage and then split into 2 equal groups, one watered to 80% field capacity (FC) (high water—HW) and one to 40% FC (low water—LW). Plants were imaged and gravimetrically watered daily using a LemnaTec Scanlyser system until the primary tiller was at Growth Stage (GS) 39–41 (Zadoks scale) which approximates to meiosis (primary tiller was tagged). Plants were then subjected to different temperature regimes as follows: 25/20, 30/25 and 35/30 °C (day/night set air temperature respectively) for 4 days, and then returned to ambient conditions within the glasshouse to complete their life cycle and ripen. At harvest the primary tiller was weighed and retained for scanning. Other parameters including the total dry weight of the plant and ears and height of the primary tiller were also collected.

### Mounting and scanning of material

For each treatment, twelve representative, fully dried primary spikes were selected for scanning and placed in plastic holders (34 mm diameter, 70 mm height). The majority of the spikes were too tall to fit in the holders so they were cut into two pieces and each scanned separately. Pieces of thermoplastic starch were used to eliminate sample movement while scanning. Sample preparation and loading into the scanner takes around 30 min per 12 samples and after this time there is no more user input. The twelve holders were loaded into the sample changing carousel of a μCT100 scanner (Scanco Medical, Switzerland). This scanner has a cone beam X-ray source with power ranging from 20 to 100 kVp (pre-set and calibrated for 45, 55, 70, 90 kVp) and a detector consisting of 3072 × 400 elements (48 µm pitch) and a maximum resolution of 1.25 µm. The samples can be positioned at different distances from the X-ray source greatly improving resolution while keeping scanning time to a minimum. Spikes were scanned with the X-ray power set at 45 kVp and 200 µA with an integration time of 200 ms. Each spike was ~ 1000 slices (51 slices per stack), 125 projections/180° were taken and a binning of 6 was used. Output images were produced with a 0.2 megapixel (512 × 512) resolution (68.8 µm/pixel) in a proprietary ISQ file type format (Scanco Medical, Switzerland).

### Computer hardware

The 3D volume was reconstructed from the projections (raw data, including flat field correction data) using proprietary software supplied with the Scanco μCT100 scanner. After 3D volume generation, the developed processing pipeline makes use of standard computing hardware. A DELL XPS desktop computer with an Intel (i7 6700k) 64 bit CPU, 64 GB of memory and an NVIDIA GPU (GTX 1080) was used.

### Computational methods and tools

A computer vision and analysis protocol was developed using a combination of the MATLAB [26] image processing toolbox and Python [27] (Additional file 1). Visualisation of processed images, stored as TIFF files, used ImageJ’s 3D viewing plugin [28] and TomViz [29]. Post-processing of results used the Scientific Python collection of software (SciPy). A full list of additional software packages can be found in supplemental table (Additional file 2: Table S1). All reconstructed 3D volumes and segmented images can be accessed at https://www.aber.ac.uk/en/research/data-catalogue/a11df174-d73d-4443-a7fd-ab5b7039df79/ [30].

### High-throughput image processing and feature extraction

A high-throughput automated pipeline was developed with the goal of reducing human input and time. Reconstructed 3D volumes were retrieved from the µCT scanner and MATLAB scripting then performed feature extraction. All the source code as well as user instructions are available from https://github.com/NPPC-UK/microCT_grain_analyser. Analysis of the resulting data is performed using Scientific Python libraries.

### Data and statistical analysis

Python scripts were used to automatically find data files and match them with information about their scanning parameters, correctly label and then compile data into tables (data frames) based on treatments, prior to analysis. Data was analysed using a collection of Scientific Python packages [27] and statistical analysis was performed using one-way ANOVA with significance calculated at p < 0.05.

## Results

### Building a robust pipeline for measuring grain morphometric data from µCT images

Computer vision approaches have been previously used to extract quantitative grain characteristics from μCT images but these tend to require high resolution images and long scan times [25]; this makes μCT expensive, hard to scale up to population size samples and technically difficult to apply to new species. In order to overcome these issues we developed a high-throughput, automated method using relatively low resolution images acquired from a bench top scanner that is easily applicable to species with diverse spike morphologies and grain sizes.

The initial test population consisted of naturally ripened dry wheat spikes (cv. Paragon) harvested from plants which had been subjected to different defined watering and temperature regimes. Dry spikes were harvested and 12 spikes per treatment were scanned at a resolution of 0.2 megapixel (512 × 512 × ~ 1000; 68.8 µm/pixel). This resolution was chosen to allow fast scan times and increase throughput while still retaining sufficient image information necessary for accurate data acquisition and analysis. Scanning time for each spike was around 40 min. We found that performing the scans at higher resolution (1024 × 1024 × ~ 2000; 34.4 µm/pixel) increased scanning time to 60 min and this did not translate into an increase in the quality of the data output for the analysed grain traits (Additional file 3: Table S2). Therefore, we chose a resolution of 512 × 512 × ~ 1000; 68.8 µm/pixel for routine scans. Higher resolutions (2048 × 2048 × ~ 4000; 17.2 µm/pixel) increased the scanning time to 3.3 h and produced a 32 Gb that was too large for routine use, but these may be useful for measurement of tissue related traits (such as thickness of the bran layer or embryo size) as discussed below.

**Fig. 1:**
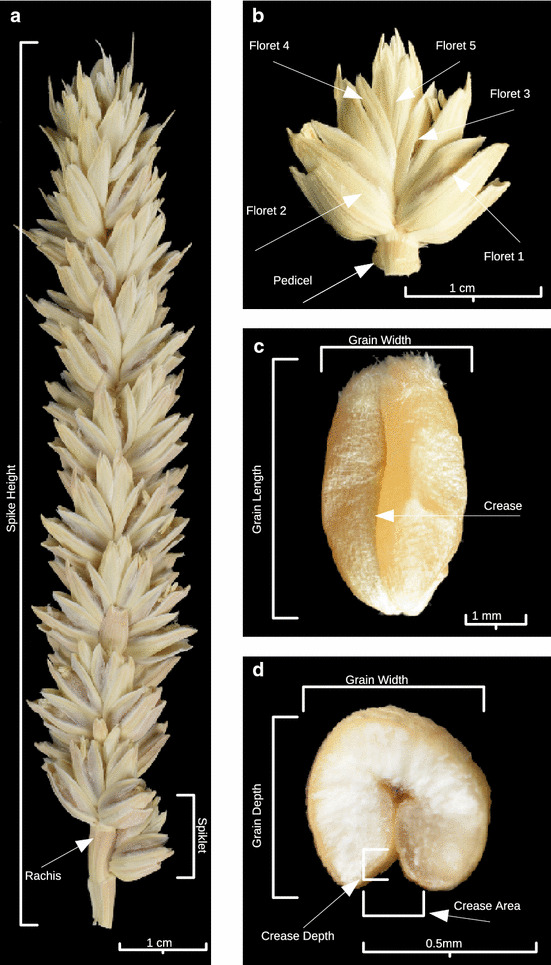
Typical wheat spike, floret arrangement and grain traits. **a** Whole spike, **b** spikelet, **c** isolated grain, **d** grain cross section. Traits measured include: total height of the spike and grain position along the spike (**a**, **b**). Measured characteristics of isolated grains included grain length and width (**c**) as well as grain depth (**d**)

Our aim was to develop a pipeline that could automatically identify and measure different grain parameters from these reconstructed volumes. The measured parameters included spike height, grain number, grain height, width and depth, grain volume and surface area (Fig. 1).

**Fig. 2:**
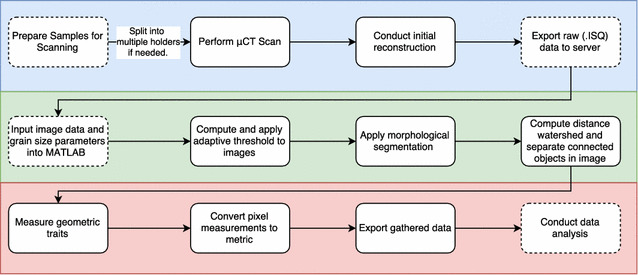
Schematic representation of the developed µCT imaging pipeline. Coloured areas represent the different stages of the method. µCT scanning and reconstruction (blue), segmentation and 3D processing (green) and feature extraction and analysis (red). Solid lines represent automated steps while dashed lines steps that require user input

A schematic representation of the pipeline used from scanning to data analysis is presented in Fig. 2. The pipeline is divided in three sections: μCT scanning and 3D volume reconstruction done by the Scanco software (Scanco medical, Switzerland); segmentation and 3D processing; and feature extraction and analysis both developed using MATLAB and Python. This pipeline is readily applicable to other plant species with varied spike and grain morphologies, and scanned at different resolutions, simply by adjusting the structured element size, the resolution and the minimum size the as detailed in Additional file 1 (setup.m). As default we set the structured element size at 5 and the minimum object size at 1000, parameters that perform well for most of the species tested. For species with very small seeds the minimum size object can be reduced.

### Segmentation pipeline

**Fig. 3:**
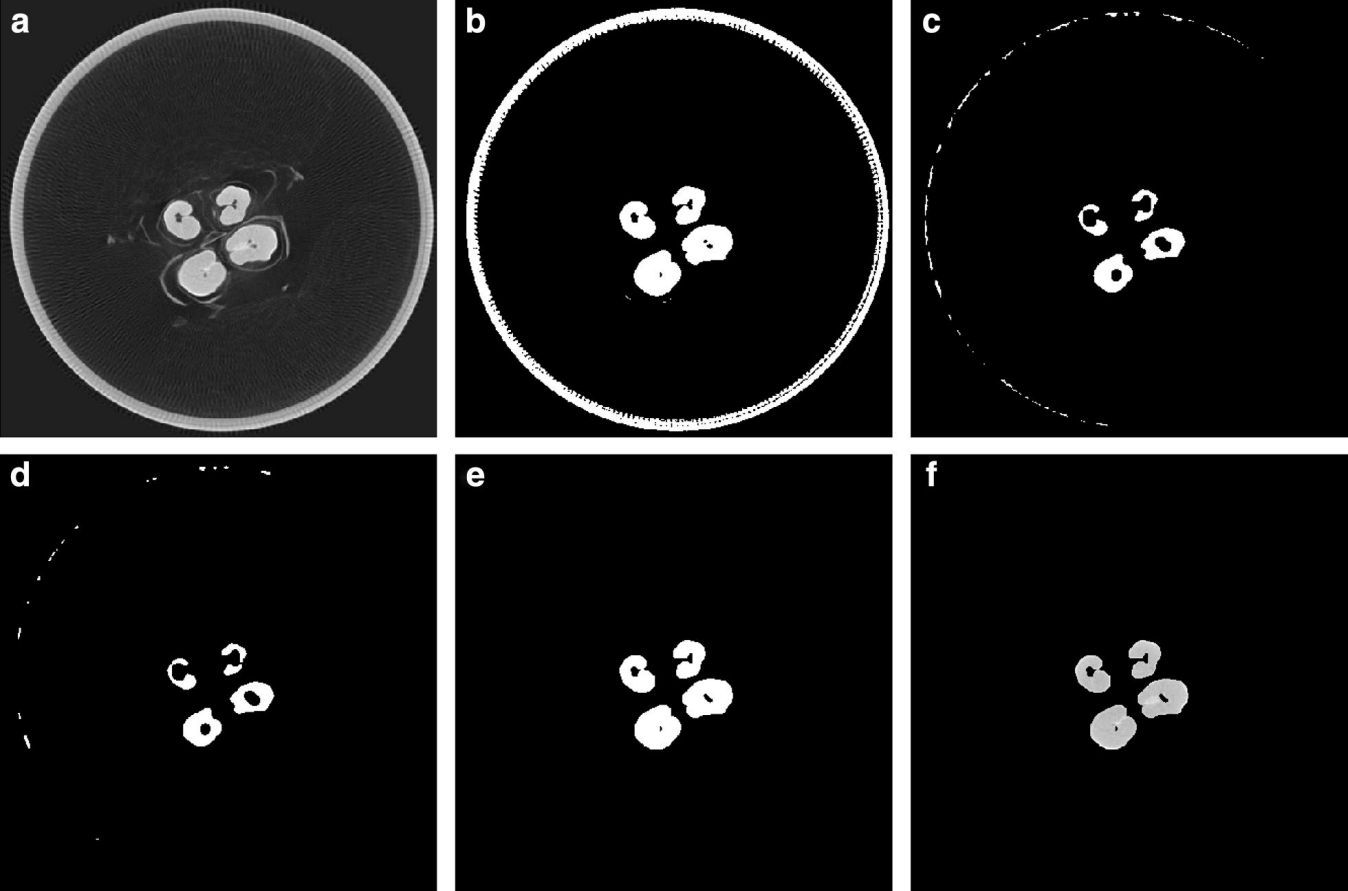
Image segmentation. **a** µCT cross section image of a typical spike in 16-bit greyscale, **b** initial thresholding using our adaptive method, **c** erosion to remove outlying objects using a disk structuring element (SE), **d** median filter applied to smooth and further segment region of interest (ROI), **e** image dilated by same SE as (**c**) and with remaining holder removed and **f** final result of this process was obtained by using (**e**) as a mask in conjunction with (**a**)

After 3D volume reconstruction, the files were exported to a data storage server. The first computational task performed was converting these data into a malleable, workable format. For this, a file-reader in MATLAB (available from [31]) was developed to generate image stacks. The 3D reconstructed volume as collected from the μCT scanner has a dual peak distribution of grey values and the use of this information has been essential in constructing an effective method for removing all non-plant material from an image [25]. To further segment the plant material of interest, we developed an adaptive thresholding method that enabled both removal of non-plant material and segmentation of grain and non-grain data (Fig. 3a, b). This developed method relied on taking a cumulative sum of grey values across all slices in a single scan and computing a minimum value for plant material. Additional file 4: Fig. S1 illustrates that material with a density value within the pink shaded area is of interest for this method.

Post thresholding morphological operations were used to perform further sanitization on the segmented images. A disk shaped structuring element (SE) [32] was used to erode the image, and then we applied a median filter before dilating. This allowed for the removal the majority of the non-target plant material and artefacts of the scanning process and obtain a segmented image (Fig. 3c, d). From this image a minimum grain size parameter is used to filter out any remaining noise, thus an image is produced containing exclusively grain material. Finding and removing the largest cluster of connected pixels throughout the 3D image allowed elimination of the outer tube used for scanning (Fig. 3e). To precisely preserve the shape of the grains along with the grey levels and ensure that there was no data loss, this segmented black and white image was used to mask the original one (Fig. 3f). After this step, the cleaned image can be used for trait extraction.

## 3D processing

**Fig. 4:**
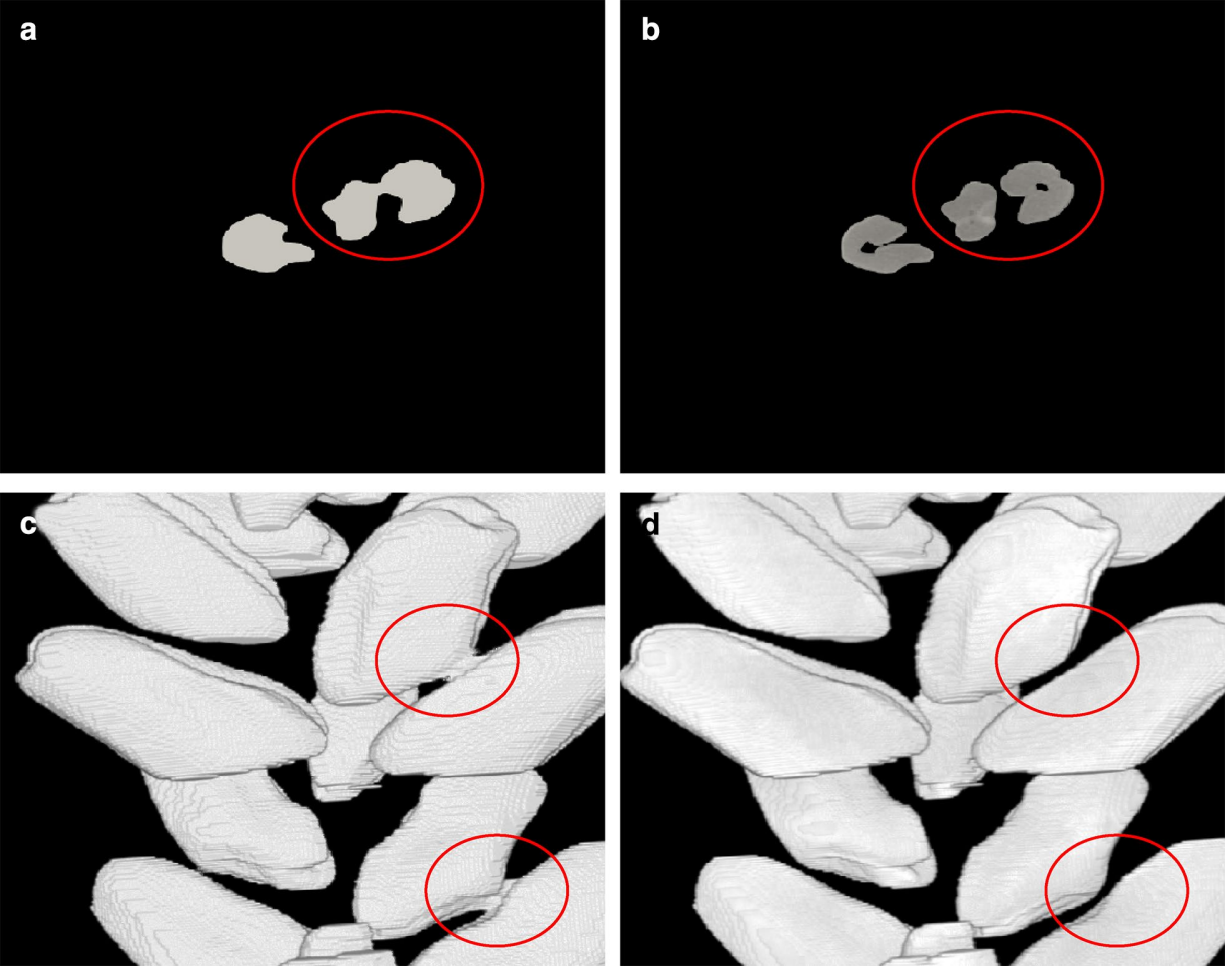
Separation of fused objects using a distance-based watershed technique. **a**, **c** Images before segmentation (red circles indicate regions of fused grains). **b**, **d** After segmentation. **a**, **b** Image cross sections, **c**, **d** 3D reconstructions

**Fig. 5:**
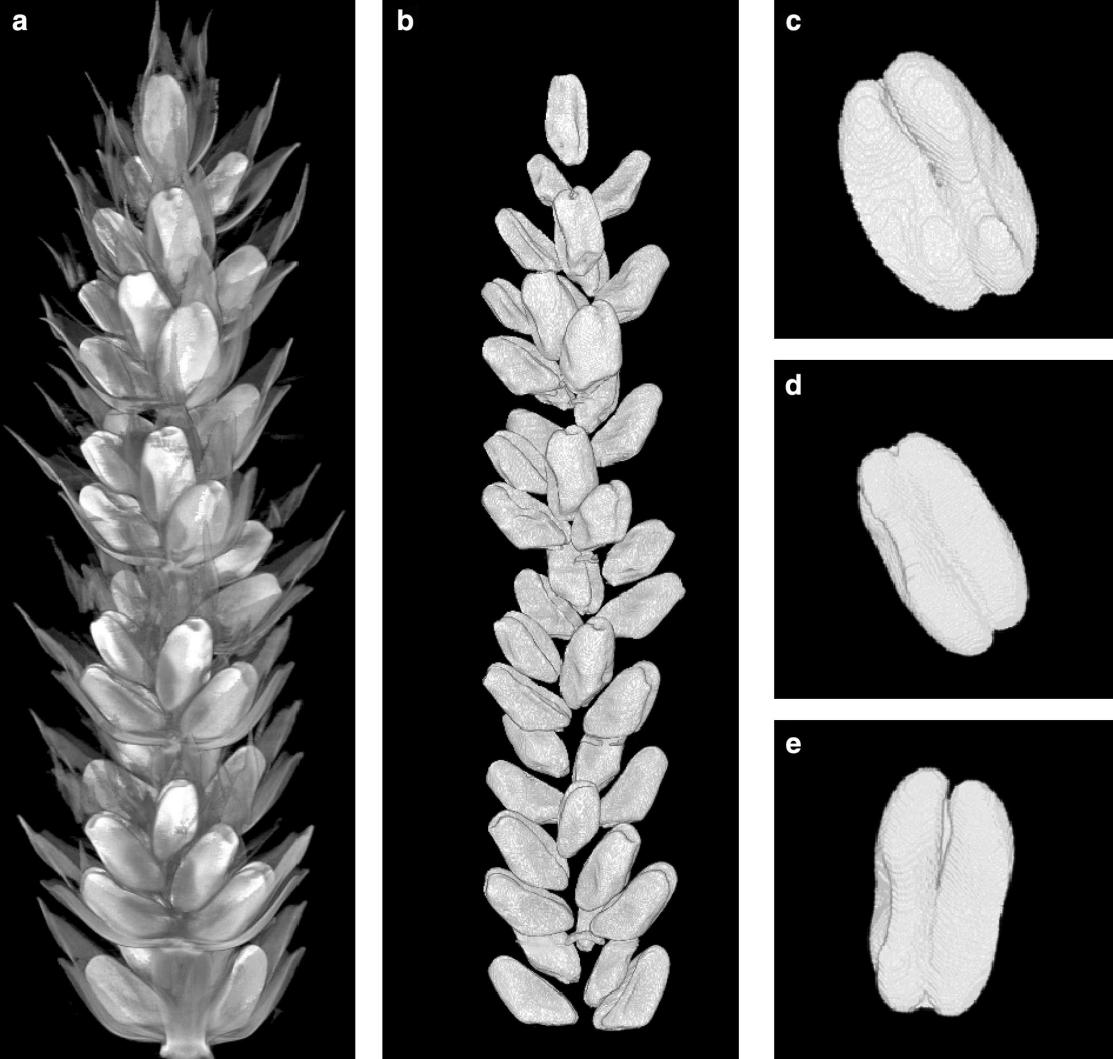
3D visualisation of images. **a** Top portion of a wheat spike before segmentation, **b** and after segmentation and **c**–**e** examples of isolated grains

There is an unavoidable trade-off between image resolution and scanning/processing times. Therefore, acquisition of low resolution images at the expense of image quality enabled us to keep scanning times at a minimum and increase the sample numbers processed. While the information needed for the morphometric analysis is still present in these lower resolution images, there is an increased possibility for objects to be artifactually fused during segmentation (Fig. 4). Such fused grains were commonly encountered (red circles in Fig. 4a, c) and would be counted and treated as single objects, thus degrading data quality and requiring extensive manual curation. To overcome this problem, a distance-based watershed technique was developed [33]. This technique was adjusted to work for 3D images by computing, for each white pixel, a distance from the nearest black pixel using a chessboard method for distance measurements [34]. With this newly computed distance map a standard watershed algorithm [35] was then applied to find dividing contour lines. This allowed for the complete separation of previously fused objects (compare red circled areas in Fig. 4a, c with b, d). After this stage the data can be used to generate 3D images as shown in Fig. 5b–e.

### Morphometric feature extraction

After segmentation and separation of all fused objects, each isolated grain was orientated by calculating the major axis of the 3D shape and was fitted to an ellipsoid in order to calculate circularity (Fig. 5c–e). The length was calculated by measuring the size of the major axis while width and depth were found by examining a cross section of each grain and taking measurements of major and minor 2D axis respectively. Volume is the complete connected-pixel count for each given grain. Surface area was calculated by adapting previously successful methods [25]. The calculation of positional data for each grain required registration at a post-processing stage, due to splitting spikes for scanning (see below).

Once compiled, grain parameters in pixels were converted to metric units by the following equation ($mm = \frac{{\left( {pixel\,*\,conversion} \right)}}{1000}$) and this data was then exported as a CSV formatted file on a per-spike basis (Additional file 5: Table S3).

### Rejoining of split spikes

Due to size limitations of the available scanner some of the samples were scanned in two portions. The positional data in the Z axis was inverted before in silico spike rejoining was performed $\left( {Z = \hbox{max} \left( Z \right) - Zi} \right)$, by incrementing top-portion scans globally by the size of the bottom scan $(Z_{i}^{t} = Z_{i}^{t} + length\left( {Z^{b} } \right)$). This could be easily generalised to 3 or more portions for longer and larger structures.

The beginning and end of a spike was calculated by locating the lowest and highest rachis points respectively in the Z axis. Detection of the rachis is done by relaxing the thresholding algorithm by 20% to detect less dense plant material.

### Estimation of accuracy

In order to evaluate the accuracy of the software in determining grain number and volume, different approaches were taken. For grain number, three separate counts were done manually on reconstructed 3D volumes. One verifier used a counting technique that involved examining each individual Z slice while the other two examined the data set in a 3D image viewer. This provided a grain count on a per spike basis that could be directly compared to the one obtained from the computer vision approach. As can be seen in Additional file 6: Fig. S2a, the software was able to accurately identify and count grains.

To confirm that no data was lost during the segmentation process, several randomly selected processed image files were compared with their original counterparts, with contour maps drawn and manually examined. This showed that there was no data loss during the segmentation process (Additional file 6: Fig. S2b).

These ground truthing results clearly demonstrate the high degree of accuracy achieved with the developed method.

We also found a strong correlation (*R* ^2^ = 0.75) between the total volume of all the grains in a spike as measured by our method and manually acquired spike weight for all the spikes (Additional file 6: Fig. S2c).

### Temperature and water regime affect grain number and grain characteristics along the wheat spike

After establishing the robustness and accuracy of the data provided by the segmentation method, we then asked whether it could provide insight into the combined effect of water regime and temperature stress on grain characteristics.

**Fig. 6:**
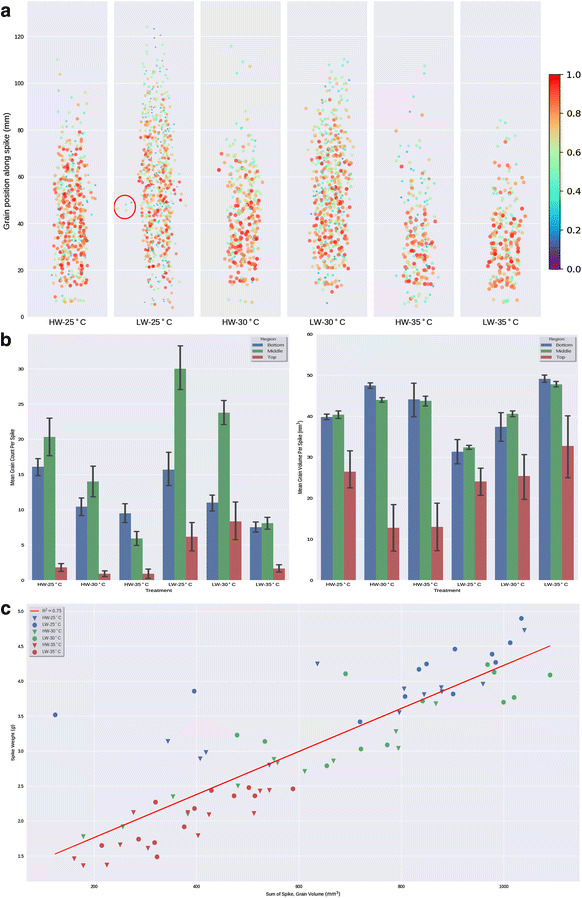
The effect of two environmental variables, water regime and temperature, on spike and grain traits. **a** Graphical representation of the total number of grains for all the spikes in a given treatment. Each circle represents an individual grain in its corresponding ZX position along the length of the spike. Colours and sizes represent the volume of the grain. Colours are normalised between 0 and 1, with 1 being the largest grain across all treatments, **b** mean grain number (left-hand panel) and grain volume (right-hand panel) per section of spike. Each spike was divided in top, middle and bottom (median spike height ± 16% was considered middle, region below that bottom and above top) and the grain number in each region calculated for each treatment and **c** relationship between grain number and grain volume per spike over all the treatments. Samples are identified by the temperature they were stressed with and HW indicates 80% FC watering while LW indicates 40% FC watering

μCT scanning confirms that grain development is differentially affected by water and heat and, in addition, that the developmental position along the spike modulates these effects. By using 3D reconstructions of whole spikes, the spatial distribution of grains along the spike is maintained and this can be overlaid with the traits measured for each grain (Fig. 6). This allows for a visual representation of how grain traits change along the spike and also how the different stresses affect those traits. A high degree of resolution was achieved in the y and z axis (Fig. 6a); this is illustrated by detection of occasional secondary spikelets that protrudes in the y-axis (Fig. 6a circled region). Generally, the middle region of the spike (in the z axis) contains more grains than the top and bottom and grains at the top of the spike are smaller (Fig. 6a, b). The effect of temperature on spike height and grain number is clearly visible with spikes grown at 35 °C showing reduced height and reduced number of grains (Fig. 6a). The major reduction in grain number occurs in the middle of the spike with the top and bottom regions being less affected (Fig. 6b). Increased temperature also leads to a reduction in grain volume at the top of the spike but this is only observed in the plants grown in a high water regime (Fig. 6b). On a per spike basis, we found an inverse correlation between average grain volume and grain number with the temperature stressed spikes harbouring fewer but bigger grains (Fig. 6c).

**Fig. 7:**
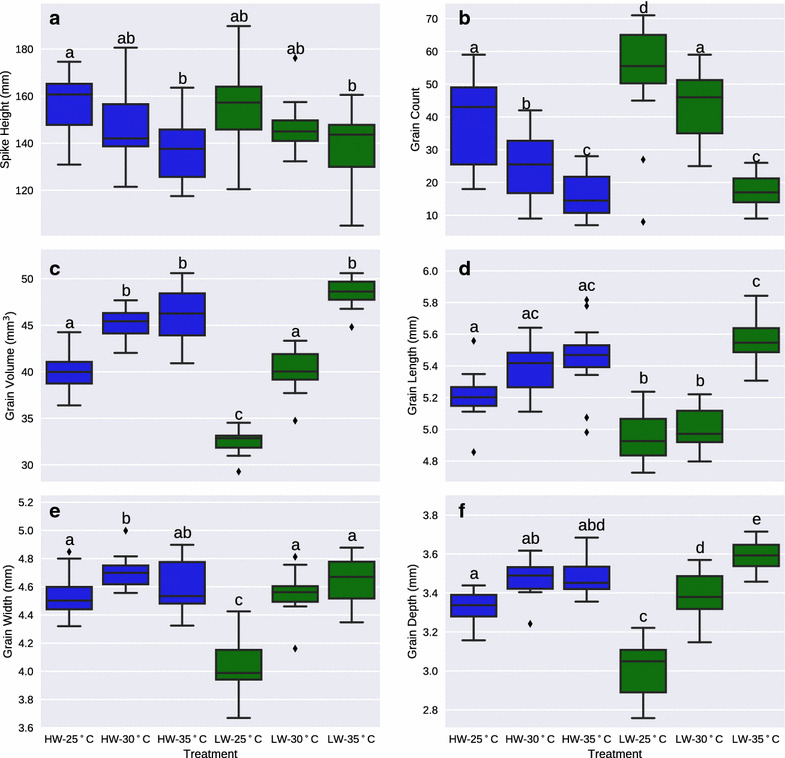
The effect of temperature (25, 30 and 35 °C) and water regimes (HW-80% FC, LW-40% FC) on wheat spike and grain traits. **a** Spike height, **b** number of grains per spike, **c** grain volume, **d** grain length, **e** grain width, **f** grain depth. Significance groups for p < 0.05 were calculated using a one-way ANOVA

Temperature had an effect on spike height with spikes being shorter in both water regimes as the temperature increased while water supply alone did not have an effect on spike height (Fig. 7a). Temperature also had a dramatic effect on grain number with temperature increases significantly reducing seed number per spike in both watering regimes (Fig. 7b). Surprisingly, we observed that the lower water regime resulted in an increased grain count at a given temperature compared to the high water although this effect was lost at the highest temperature (Fig. 7b).

Grain volume was also strongly influenced by growth conditions, shown by a general increase of individual grain volume with increased temperature (Fig. 7c). As noted for Fig. 6c, the observed grain volume increase is associated with a decrease in grain number for a given treatment (Fig. 7b, c). The observed increase in volume is a result of a general increase in grain length, width and depth (Fig. 7d–f).

## Discussion

Food security has been identified as one of the largest challenges being faced in the world today [36]. Globally we have become increasingly dependent on a select handful of plant species and as a result an increasing importance is being put on research of these crops [37]. In many crops yield is dependent on the stability and uniformity in grains (shape, size and yield) and this has been the target of breeding programmes. The current challenge is to develop methods able to measure grain traits on a large scale in a quick and robust manner.

In this study we demonstrate that X-ray micro-computed tomography (μCT) can provide non-destructive, quantitative data on the environmental impact of stress on grain traits within their normal developmental context. Moreover this can be done quickly, accurately, and is scalable to large sample sizes with minimal user intervention.

### μCT as the method of choice for spike and grain trait analysis

There is a scarcity of organ-level imaging approaches that lend themselves to rapid quantitative measurements suitable for in-depth physiological or genetic dissection and modelling. Light and electron microscopy are widely used but they provide limited information and tend to be labour-intensive to produce [38]. Other techniques using conventional cameras that rotate around the subject can also generate accurate 3D model but do not provide information about the internal structure of plant material [39, 40]. As the organs of interest are often embedded within other tissues, the techniques described above require the manual removal of the surrounding tissue. This can be time-consuming and spatial/developmental information is easily lost. Methods have been designed to allow automated removal of grains from the spike while retaining positional information, but these are highly specialized and expensive instruments [41].

These limitations can be largely overcome by μCT. μCT has traditionally been used, to great effect, in medical imaging, and its applications in plant science have increased over the last few years [17–25]. Methodologies developed in the medical field have been applied to wider biological studies, for example techniques used to model regions of the human heart [42, 43] have more recently been used to examine seed anatomy [18] and methods used to study metamorphosis in insects [44], modified to track root development in soil and non-destructive floral staging [19, 45].

One of the critical advantages of μCT imaging is that positional information of organs and tissues is preserved and can be analysed. This is extremely important when looking at changes throughout development and variation in grain traits within each spikelet or along the length of a spike is a good example. Imaging of internal tissues and organs without dissection is also possible, although this will require scanning at higher resolutions. Thus non-destructive imaging of the bran layer and the embryo, both of which are economically important traits, could be further developed and scaled for breeding and quality control applications. Finally, detailed study of specific 3D grain parameters such as circularity, surface area and crease volume which are agronomically relevant are also made possible by this method.

### Constraints of the scanning and image analysis methodology

Underlying the increased use of μCT in plant biology has been the development of more affordable small, and even benchtop, μCT scanners with sample loading carousels more suitable for larger sample numbers. However, their use necessitates a number of trade-offs between sample number, size and data quality. For example, the loading carousel imposes physical limitations on the size of individual samples and we had to divide many spikes. To re-integrate measurements taken from separate portions of the same spike, we identified conjoining points along the rachis of each spike and rejoining images was introduced as an additional processing step. Further issues can emerge from the use of a fixed X-ray beam which rotates the subject to obtain a 360° image. This gives opportunity for movement during scanning resulting in minor image distortion. To limit movement, scanning material was held in place using thermoplastic starch that, although visible in the scan, can easily be removed by the application of morphological filters during image processing. The time required to produce and reconstruct high resolution scans represents, perhaps, the most serious bottleneck for routine grain analyses. For a wheat spike this can take several hours using typical hardware. To address this, the scans were performed at the lower resolution of 0.2 megapixels (512 × 512) rather than much higher resolutions used in previous studies, for example 5 megapixels (2048 × 2048) and larger is often used [19]. This also reduced the output file size on average by a factor of 16. The trade-off for this lower resolution was the decrease in spatial accuracy resulting in the incorrect joining of juxtaposed objects; this was rectified during the segmentation process.

### Development of a robust computer vision pipeline

During our initial attempts to analyse the data produced through μCT we discovered that there was a lack of software that could handle the volume of the data and implement modern computer vision algorithms easily and was well-suited to high-throughput automation. VGStudio Max, a commercially licensed software package, and BoneJ, a free and open source software package, are often used in biological and medical science for image analysis and visualisation [19, 46, 47]. However they require human interaction on a per image basis. While this level of interaction is justifiable for high value subjects (i.e. in a medical context), the scale required for crop biology demands minimal intervention.

This prompted us to design and create a new computer vision based methodology. Our aim was to develop an entirely adaptable system which we could build upon in future, and robust enough to work with a multitude of grain shapes and sizes. The MATLAB [26] scientific programming language and environment provided a widely available professional platform that has closely related open-source alternatives (Octave [48]) that can be used to implement our method, albeit with reduced functionality (some of the watershedding techniques are not yet implemented in Octave).

### Suitability for grain trait analysis

As a proof of principle, the developed methodology was used to study the effect of temperature and water regime on spike development and grain traits on a population of wheat plants. We found that temperature differentially affects grain formation along the spike with the middle of the spike being more sensitive to the stresses. Recent studies have shown that there are two discrete developmental stages where the spike is more sensitive to temperature: early booting when meiosis is occurring and anthesis [8–10]. Floret development along the spike is asynchronous [12] it is thus tempting to speculate that the florets in the middle were at a temperature-sensitive stage when the stress was applied. In agreement with previous reports [9] we also found an inverse relationship between grain number and grain volume across treatments. While high temperature and high water regime caused a decrease in the number of grains per spike, the average volume of grains increased, partially compensating for grain loss. It should be noted that the low water plants were slightly ahead in terms of spike development when the heat stress was applied and this could explain why in these plants’ temperature has a less detrimental effect on grain number per spike. Despite suggestions that grain height, width and depth are affected by independent sets of genes [49], our data indicates that the response of these traits to different growth conditions are highly correlated. It will be informative to extend these studies to diversity and mapping populations to explore how changes in spike architecture and grain traits in response to multiple stresses are genetically controlled.

**Fig. 8:**
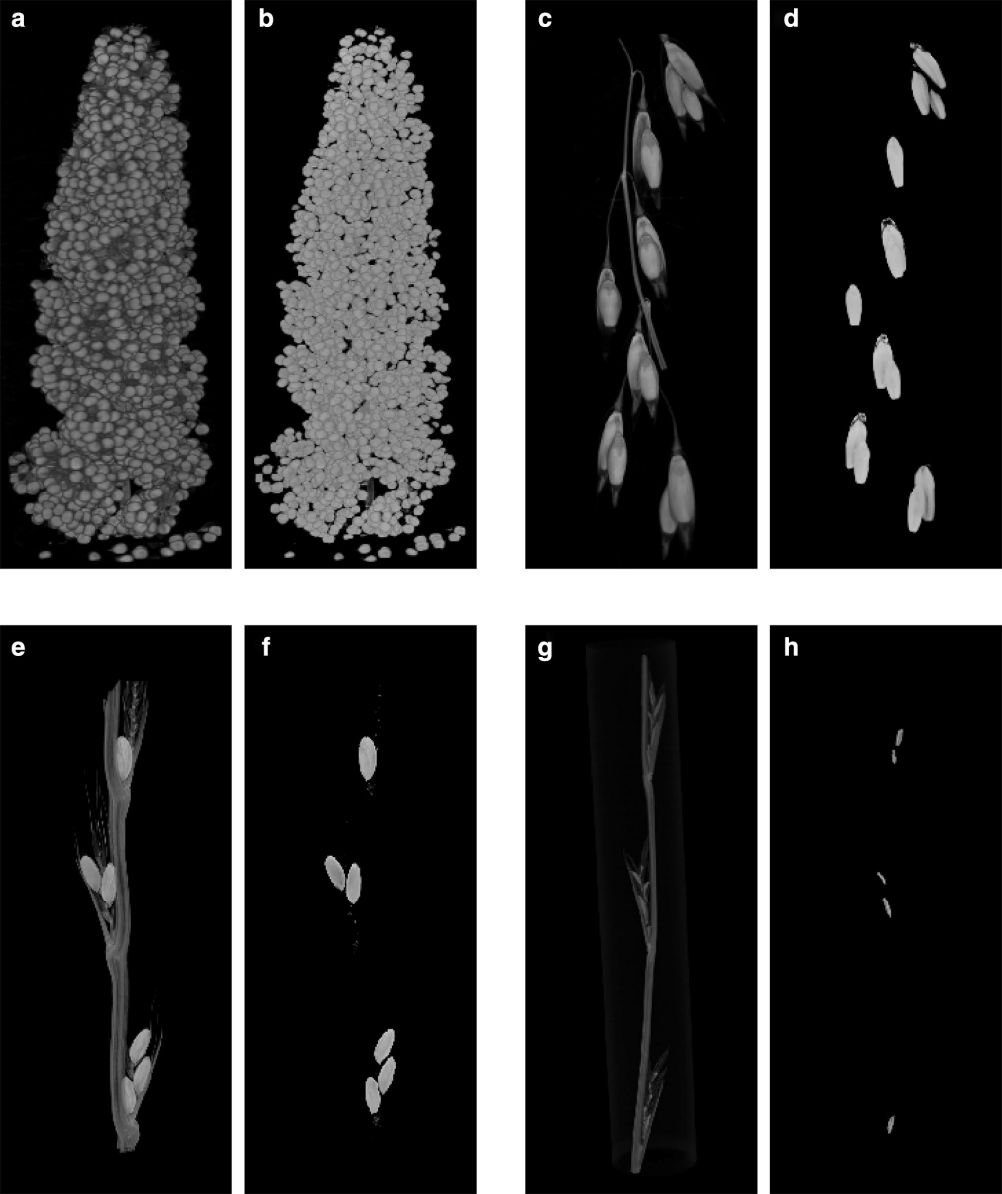
Evaluating methodological versatility: 3D reconstructions of µCT images. **a**, **b** Foxtail millet (*Setaria italica*), **c**, **d** oats (*Avena sativa*), **e**, **f** darnel ryegrass (*Lolium temulentum*) and **g**, **h** ryegrass (*Lolium perenne*). **a**, **c**, **e**, **g** Pre-segmentation images and **b**, **d**, **f**, **h** post-segmentation images

Finally, to demonstrate the wider applicability of the method, we examined different species (foxtail millet, oats, darnel ryegrass and ryegrass) that illustrate a diversity of inflorescence and grain morphologies, from the dispersed open panicle structure of oats to the very congested structure of millet which has numerous small grains packed together (Fig. 8). In all cases, simply by adjusting two parameters (the structuring element size, and minimum grain size) our method identified the grains and performed grain feature extraction accurately (Additional file 7: Table S4).

### Challenges and future perspectives

Grain uniformity is of economic value in many cereals and is an active breeding target. Grain size changes not only along the spike but also within each spikelet. Relating the position of an organ in physical space to its position in developmental space is a wider challenge, well-illustrated by the contrast between oats and millet but also applicable to other grasses. Besides the grain morphometric traits, the use of μCT may also provide a handle on more difficult to measure traits like crease volume and thickness of the bran layer. Both of these traits are commercially important and determine grading of grains for the milling industry, but are extremely difficult to measure. Embryo size in the seed is also thought to be important in determining seedling growth and final biomass of the plant, but again this is difficult to measure in a non-destructive way [50].

The challenge now is to develop more advanced computing methods that are able to detect and measure these highly complex and variable traits. Recent developments in computer vision methods and machine learning modelling should prove to be very useful for this purpose.

## Conclusions

X-ray μCT offers advantages over traditional techniques to measure morphometric traits in a non-destructive, non-invasive way. Here, we show that fast, relatively low resolution scans, combined with refined segmentation techniques and 3D feature extraction are effective in providing robust and accurate results with minimal user intervention. We used this methodology to study the effect of abiotic stresses on wheat spike and grain morphology, and also show that the method is applicable to other economically important grasses. When applied to whole populations, this methodology could be extremely informative and be used in targeted breeding programs.

## Additional files

**Additional file 1.** MATLAB Image processing pipeline. This file contains all image processing code and instructions explaining input and output as well as usage of our method.

**Additional file 2: Table S1.** Additional software packages used in this study.

**Additional file 3: Table S2.** Dataset obtained for the same wheat spike scanned at different resolutions as indicated. No significant difference for p < 0.001 was found for traits measured either in the 68.8 µm/pixel or the 34.4 µm/pixel 3D reconstructed volumes.

**Additional file 4: Fig. S1.** Bimodal distribution of grey values. Histograms for 4 different scans are shown. Grey values in the pink shaded region were used for segmentation.

**Additional file 5: Table S3.** Dataset used. Tables contain all the collected data for grain and spike parameters.

**Additional file 6: Fig. S2.** Ground truthing data. (a) Comparison of grain counts obtained by the method and manual counts done by 3 independent people. Bars represent average ± SD of the 3 counts. (b) Processed image file with segmented out region in green, overlaid with the original image to show that no grain data is lost. (c) Correlation between manual acquired spike weight and grain volume determined by the developed method.

**Additional file 7: Table S4.** Grain trait data for foxtail millet (*Setaria italica*), oats (*Avena sativa*), darnel ryegrass (*Lolium temulentum*) and ryegrass (*Lolium perenne*).

## Abbreviations

µCT: micro computed tomography
MRI: magnetic resonance imaging
PET: positron emission resonance
NMR: nuclear magnetic resonance
3D: three-dimensional
2D: two-dimensional
L: litre
FC: field capacity
HW: high water
LW: low water
mm: millimetre
kVp: peak kilovoltage
µm: micrometres
µA: microamps
ms: miliseconds
GPU: graphical processing unit
ANOVA: analysis of variance
p value: probability value
cv: cultivar
SE: structuring element
ROI: region of interest
CSV: coma separated value
