# Supplementary material for: Non-destructive, high-content analysis of wheat grain traits using X-ray micro computed tomography
Source: Plant Methods. 2017 Nov 1;13:76. doi: 10.1186/s13007-017-0229-8 (PMC5664813; doi:10.1186/s13007-017-0229-8)
Supplement: Supplementary file 1 — Additional file 1. MATLAB Image processing pipeline. This file contains all image processing code and instructions explaining input and output as well as usage of our method. [file 13007_2017_229_MOESM1_ESM.zip › Additional file 1/instructions.pdf]

Usage Instructions for Micro-CT Plant Images

Nathan Hughes (nah31@aber.ac.uk)

July 28, 2017

Contents

1 Usage **2**
1.1 Setup variables 2
1.2 Running 2

2 Files and Functions **2**
2.1 cleanWheat 2
2.2 countGrain 2
2.3 filterSmallObjects 2
2.4 imSurface 2
2.5 processDirectory 2
2.6 rdir 2
2.7 readISQ 2
2.8 segmentRachis 3
2.9 watershed3D 3
2.10 writeTif 3

3 Output **3**

1 Usage

Usage of this software is straightforward. Inputting a directory, a voxel size and a minimum size of expected grain objects will output and write grain statistics and image to file.

1.1 Setup variables

A brief setup of environment variables are required, this is an example:

```
voxelSize = 68.8; % or whatever micro-meter to voxel ratio was used in scanning
minimumGrainSize = 10000; % a minimum grain size of interest
structEleSize = 5; % a size of structuring element to use for morphological operations

% Every folder in CT-Scans folder and every ISQ file in them
directory = '/home/files/CT-Scans/*/*.ISQ';
```

1.2 Running

Running the program is as simple as calling the processDirectory function.

```
% Will process all files found by rdir function
processDirectory(directory, structEleSize, voxelSize, minimumGrainSize);
```

2 Files and Functions

2.1 cleanWheat

cleanWheat is a function which takes as input a filename location on disk of an ISQ raw image, it processes it and outputs a binary 3D image and a greyscale 3D image which has been cleaned and segmented.

2.2 countGrain

countGrain takes cleaned image data, separates each identified grain and computes statistics on a grain-per-grain basis. It returns two statistics objects, one with raw pixel data counted and another with computed metric values.

2.3 filterSmallObjects

filterSmallObjects attempts to remove all objects which are smaller than the specified parameter during setup. This uses pixel size **not** metric sizes for this.

2.4 imSurface

imSurface is a library originally by David Legland. It measures the surface area in pixels of a 3D object.

2.5 processDirectory

processDirectory is the main controlling function of this software, it moves image data around from function to function, gathers image results/measurements and saves it to disk from here.

2.6 rdir

rdir is a function which recursively finds files, it is used to find files in sub-directories by using the '*' wildcard in the directory name parameter.

2.7 readISQ

readISQ originally developed by Johan Karlsson, we have modified it to make speed increases and added specific slice loading, this helps for increased speed when processing larger images

2.8 segmentRachis

segmentRachis finds locations of nodes along the rachis of spikes of wheat, oats etc. Use of this data is primarily for locating joining points of split scans.

2.9 watershed3D

watershed3D incorporates traditional watershedding techniques and has adapted them to work in 3D. It also makes use of modernised distance-based watershed methods, by way of chessboard distance technique.

2.10 writeTif

writeTif writes image stacks to disk as TIF formatted files.

3 Output

From successful running of this software output will be:

- A statistics of grains CSV with metric values
- A statistics of grains CSV with raw values
- A TIF file of the segmented image
- A statistics file of the rachis top and bottom points.
- A folder of 2D cross sectional images, for each grain
- A folder of 3D TIF files, each a individual grain

The output folder should look similar to this:

Name	Size
C0001375.ISQ.csv	7.3 kB
C0001375.ISQ	533.7 MB
C0001375.ISQcleaned.tif	267.3 MB
C0001375.ISQ-grains	37 items
C0001375.ISQ-grain-stacks	37 items
C0001375.ISQ-raw_stats.csv	3.4 kB
C0001375.ISQ-rstats.csv	32 bytes
