# Supplementary figures and images for: Non-destructive, high-content analysis of wheat grain traits using X-ray micro computed tomography
Source: Plant Methods. 2017 Nov 1;13:76. doi: 10.1186/s13007-017-0229-8 (PMC5664813; doi:10.1186/s13007-017-0229-8)

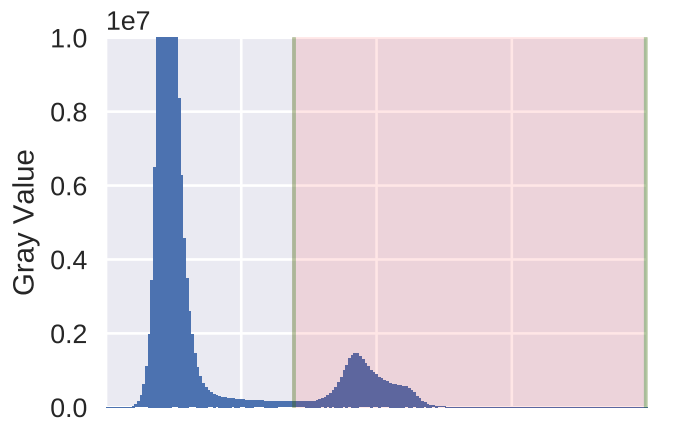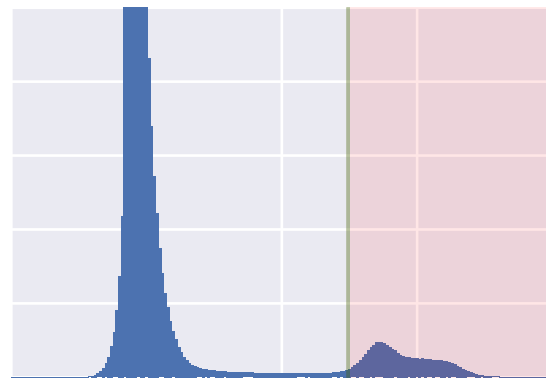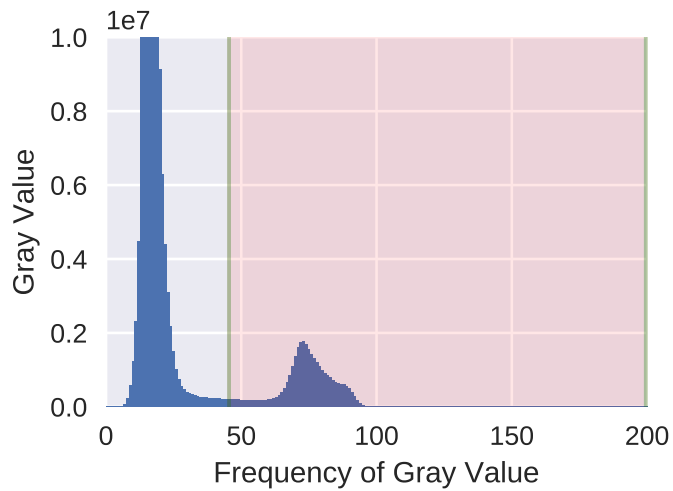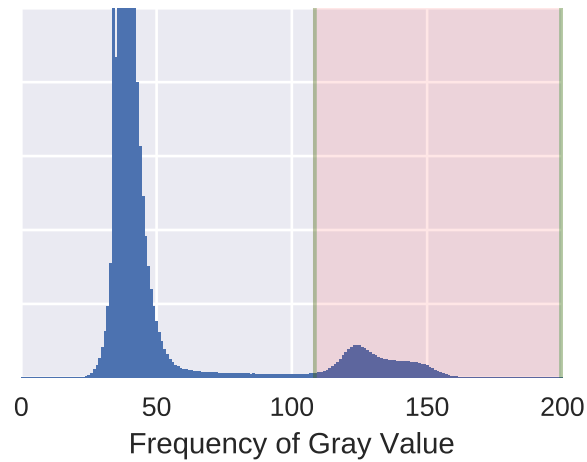

Supplement: Supplementary file 4 — Additional file 4: Fig. S1. Bimodal distribution of grey values. Histograms for 4 different scans are shown. Grey values in the pink shaded region were used for segmentation. [file 13007_2017_229_MOESM4_ESM.pdf]

(a)

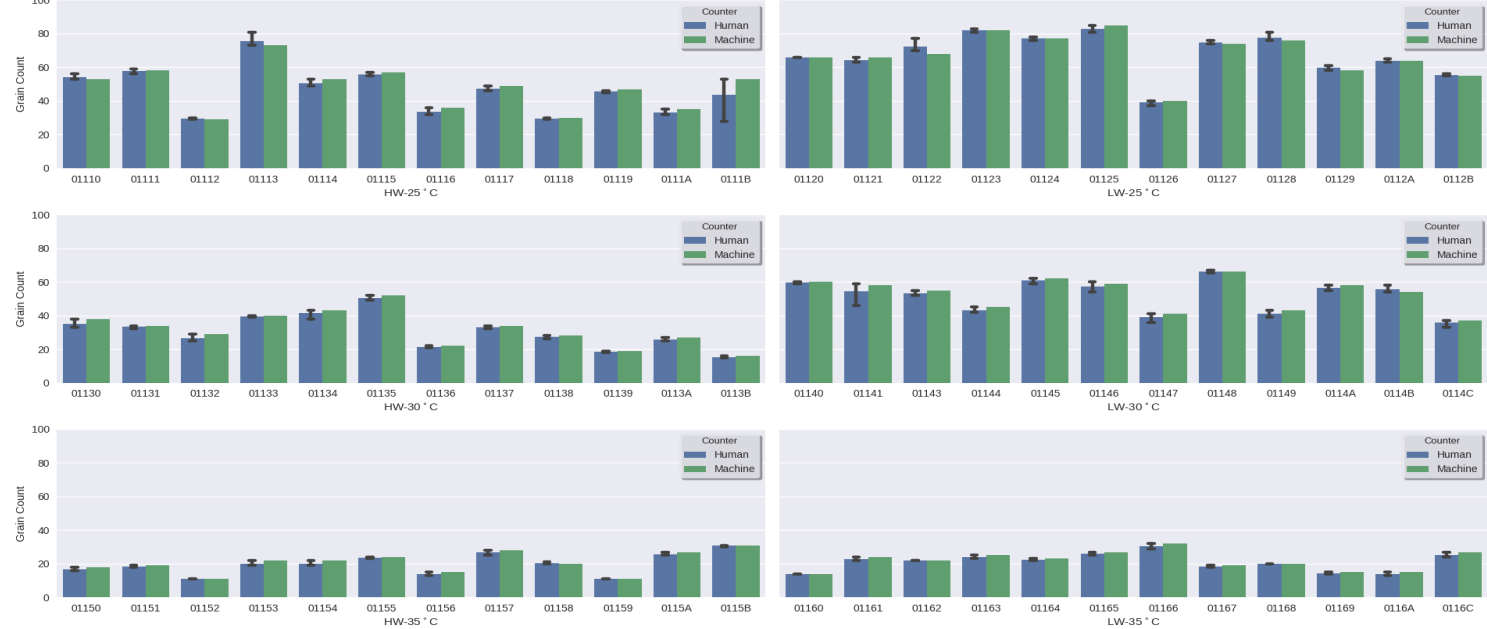

(b)

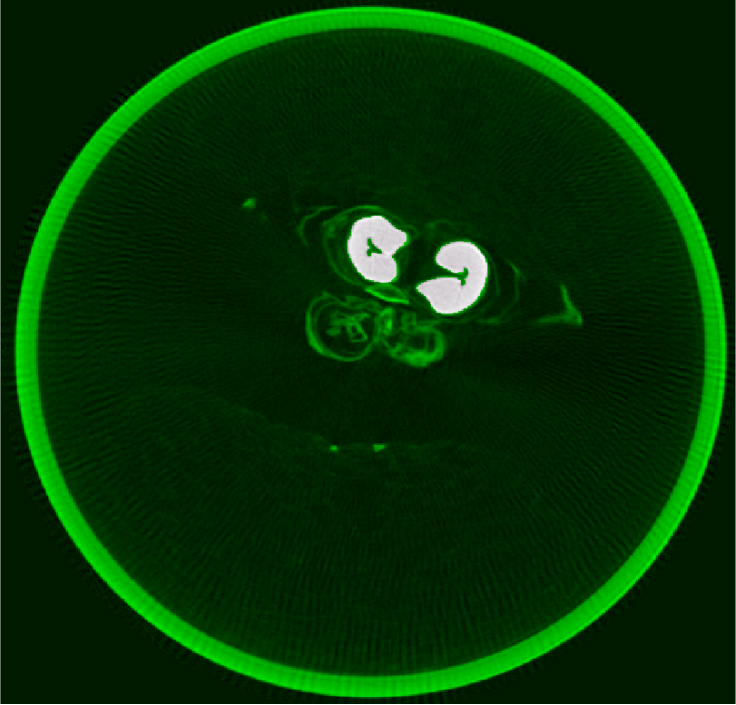

(c)

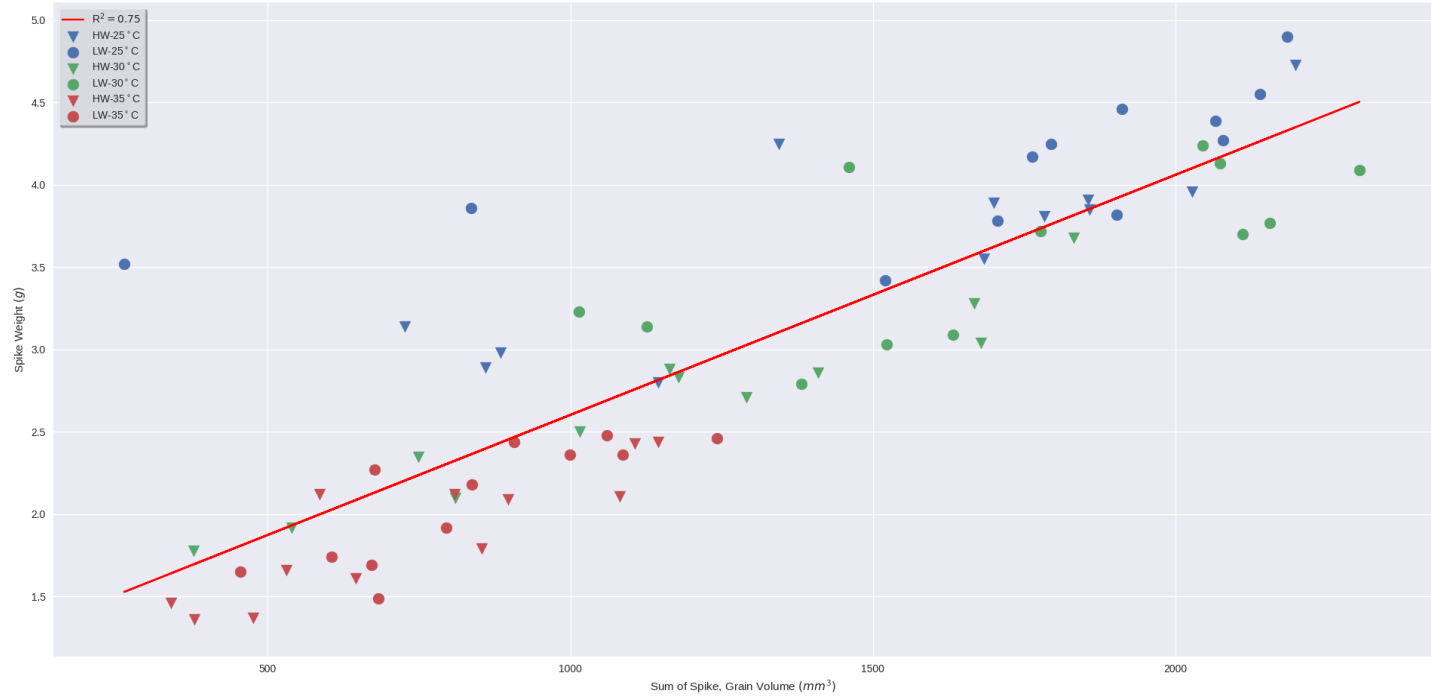

Supplement: Supplementary file 6 — Additional file 6: Fig. S2. Ground truthing data. (a) Comparison of grain counts obtained by the method and manual counts done by 3 independent people. Bars represent average ± SD of the 3 counts. (b) Processed image file with segmented out region in green, overlaid with the original image to show that no grain data is lost. (c) Correlation between manual acquired spike weight and grain volume determined by the developed method. [file 13007_2017_229_MOESM6_ESM.pdf]
